# Assessing the survival time of women with breast cancer in Northwestern Ethiopia: using the Bayesian approach

**DOI:** 10.1186/s12905-024-02954-y

**Published:** 2024-02-15

**Authors:** Chalachew Gashu, Aragaw Eshetie Aguade

**Affiliations:** 1 Department of Statistics, College of Natural and Computational Science, Oda Bultum University, Chiro, Ethiopia; 2 Department of Statistics, College of Natural and Computational Science, University of Gondar, Gondar, Ethiopia

**Keywords:** Breast cancer, Survival time, Bayesian, Integrated nested Laplace approximation

## Abstract

**Background:**

Despite the significant weight of difficulty, Ethiopia's survival rate and mortality predictors have not yet been identified. Finding out what influences outpatient breast cancer patients' survival time was the major goal of this study.

**Methods:**

A retrospective study was conducted on outpatients with breast cancer. In order to accomplish the goal, 382 outpatients with breast cancer were included in the study using information obtained from the medical records of patients registered at the University of Gondar referral hospital in Gondar, Ethiopia, between May 15, 2016, and May 15, 2020. In order to compare survival functions, Kaplan-Meier plots and the log-rank test were used. The Cox-PH model and Bayesian parametric survival models were then used to examine the survival time of breast cancer outpatients. The use of integrated layered Laplace approximation techniques has been made.

**Results:**

The study included 382 outpatients with breast cancer in total, and 148 (38.7%) patients died. 42 months was the estimated median patient survival time. The Bayesian Weibull accelerated failure time model was determined to be suitable using model selection criteria. Stage, grade 2, 3, and 4, co-morbid, histological type, FIGO stage, chemotherapy, metastatic number 1, 2, and >=3, and tumour size all have a sizable impact on the survival time of outpatients with breast cancer, according to the results of this model. The breast cancer outpatient survival time was correctly predicted by the Bayesian Weibull accelerated failure time model.

**Conclusions:**

Compared to high- and middle-income countries, the overall survival rate was lower. Notable variables influencing the length of survival following a breast cancer diagnosis were weight loss, invasive medullar histology, comorbid disease, a large tumour size, an increase in metastases, an increase in the International Federation of Gynaecologists and Obstetricians stage, an increase in grade, lymphatic vascular space invasion, positive regional nodes, and late stages of cancer. The authors advise that it is preferable to increase the number of early screening programmes and treatment centres for breast cancer and to work with the public media to raise knowledge of the disease's prevention, screening, and treatment choices.

## Introduction

Uncontrolled cell growth and dissemination is a hallmark of the cancer group of disorders. There may be fatalities if the spread is not stopped. In contrast to 12.7 million and 7.6 million, respectively, in 2008, GLOBOCAN 2012 estimates that 14.1 million new cancer cases and 8.2 million cancer-related fatalities transpired in 2012 [1]. The most common cancer in women worldwide is breast cancer, with over 1.7 million new cases and 522,000 deaths from linked causes in 2012 [2]. In Africa, cancer is the third-most common cause of death and a hazard to public health. It is a set of illnesses characterized by the body's aberrant cells growing and spreading out of control [3]. Around 7% of deaths in Ethiopia are caused by cancer [4]. Around 60,960 new cases of cancer are diagnosed each year, and over 44,000 people die from the disease. Breast cancer (BC) (30.2%), cervical cancer (13.4%), and colorectal cancer (5.7%) are the three cancers that affect Ethiopia's adult population the most frequently [4, 5].

Previous research showed that tumor size, metastasis, late stage, lymph vascular space invasion, metastases number, endocrine therapy are predictors of survival time of breast cancer outpatients [6, 7]. Young age, advanced stage at diagnosis, positive lymph node status, tumor sizes 3 and 4, positive lymph node status, and the presence of hormone receptor-negative status are other predictors of survival [8].

Breast cancer risk is increased by exogenous hormone exposure from things like oral contraceptives, HRT, and dietary fat intake [9, 10]. Approximately 70% of females who acquire breast cancer do not have any recognized risk factors, despite the belief that all of these risk factors exist [11].

Breast cancer accounts for 34% of all female cancer cases in Ethiopia, according to the Addis Ababa cancer registry data, while breast cancer accounts for 16% of cases [12]. In emerging and low-income nations, the disease continues to be a public health concern (LMIC) [13]. According to the information provided above, many of the women in Ethiopia are at high risk of developing breast cancer. Researching the survival rates of breast cancer outpatients is crucial for all of the aforementioned causes. We employed parametric survival models from the Bayesian approach in this journal. Breast cancer research has, however, been conducted in some cases. The majority of them were done in Ethiopia and used logistic regression to determine knowledge [14], screening procedures using logistic regression [15], and Cox proportional hazard regression analysis to determine factors that determine how long breast cancer patients will live after diagnosis [2].

The study did not emphasis the weight of outpatients with breast cancer, which is one of the major variables determining prognosis. In a large population-based cohort study of women with stage 0-IV breast cancer, we measured weight change from diagnosis to about 18 months post-diagnosis and examined its associations [16]. Furthermore, logistic regression does not account for censoring observation, i.e., it does not hold for time-to-event data, therefore, these statistical methods are unable to account for the hospital patient survival rate. In addition to the Cox regression model, other parametric models have also been employed to examine the survival distribution of outpatients with breast cancer, including exponential, log-logistic, and Weibull log-normal models [17]. The parametric survival models might be more suitable to describe the survival data if it is possible to identify the distribution of the survival time [18]. The accelerated failure time (AFT) models, such as Weibull, exponential, log-logistic, and log-normal, have a more realistic interpretation and produce more meaningful findings in comparison to the Cox proportional hazards (Cox-PH) model [19].

Parametric survival models are crucial to Bayesian survival analysis since many real-world Bayesian studies use parametric AFT models and provide computational advantages by employing the Markov Chain Monte Carlo (MCMC) approach. The observed data is fixed, and the model parameters are random according to the Bayesian approach. Using the prior probability distributions is an effective way to incorporate information from past studies and lessen confounding [20].

By applying the Bayes theorem to the data, the Bayesian techniques integrate information from the data with unbiased prior knowledge [21]. The time-consuming nature of approximating the posterior and the convergence problem are two downsides of the MCMC techniques [22, 23]. In contrast to other estimation techniques, the Bayesian approach with the Integrated Nested Laplace Approximation (INLA) method of estimation quickly and accurately approximates the posterior marginal distributions of the model's parameters through the clever application of Laplace approximations and sophisticated numerical techniques that benefit from sparse matrices' computational advantages [24].

Therefore, considering its advantages was the primary factor in the decision to use Bayesian analysis on the breast cancer data set in this study. We made the decision to examine the breast cancer data set using Bayesian parametric survival models, applying the INLA approach, because breast cancer is a significant problem in countries with hospital-based care and gaps have been found in most research. As a result, the goal of this study is to present fundamental knowledge about the variables that have a significant impact on the estimated survival time of outpatients with breast cancer as well as the best parametric survival models for the analysis of the breast cancer data set. In this study, the best parametric survival models for a breast cancer data set are determined, prognostic markers for the survival of breast cancer patients are identified, and the Bayesian accelerated failure time models are investigated using the INLA method.

Examining the survival rates of breast cancer outpatients is one way to discover risk factors for mortality and address the health issue in society. The results of this study may also be used to increase public knowledge of the factors that contribute to the death of outpatients with breast cancer. Furthermore, it allows us to communicate the results with the Ethiopian Ministry of Health in order to assist policymakers in raising public awareness of the factors that raise the risk of breast cancer-related death, which may be avoided and treated if it is recognised early and given the appropriate care.

## Methodology

### Data description

#### Study area and target population

Data from the University of Gondar referral hospital, 720 kilometres northwest of Addis Abeba in Ethiopia's Amhara National Regional State, were used to conduct the study [25]. The entire outpatient breast cancer population at UOGRH who had been registered for 4 years, from May 15, 2016, to May 15, 2020, was the study's target population.

#### Inclusion criteria

Patients who started breast cancer treatment at the University of Gondar referral hospital between May 15, 2016, and May 15, 2020, and who had at least two follow-up visits for prescription refills at the department clinic were included in this study. The enrolled patients suffered from co-morbidities, and they were administered any treatment regimen.

#### Exclusion criteria

Patients who are visiting a clinic for prescription refills while receiving therapy for breast cancer, those who haven't been there for at least 2 follow-up visits, and those who are outside of the study period weren't included in the study.

#### Study design and sample size

A retrospective study design was used in this investigation. Without prior planning for the demands of the study, data are collected from UoGRH. Only a few outpatients with breast cancer were encountered by the researcher. As a result, the researcher decided against using a sample method for this investigation. This study includes all hospitalized breast cancer patients who met all inclusion criteria and were admitted between May 15, 2016, and May 15, 2020.

#### Data source and data collection procedure

The study's data source was secondary data. The University of Gondar, College of Natural & Computational Science ethical approval committee, in Gondar, Ethiopia, has granted authorization (reference number: 02/03/976/10/2014). Then, using a checklist (data extraction form), the lead investigator and a trained enumerator collected secondary data based on data already present in the hospital.

*Starting time* the interval's beginning time (in months). The study would take into account survival data going back to the patient's initial day of therapy, which is the day of diagnosis for outpatients with breast cancer.

*Ending time:* the period of time (in months) when the event took place, when the outpatients with breast cancer died or were no longer being followed up on May 15, 2020 (the study's end date), expressed in months. This indicates that the censoring type used on survival data is right-censored.

#### Variables in the study

The time in months between the time of diagnosis and the occurrence of an event (such as "lost to follow-up," "death," "stopped," "dropped out," or "transferred to other health centres or hospitals") was the response variable for outpatients with breast cancer. Patients had follow-up visits for prescription refills at the department clinic every 6 months. The event of interest for this study was death. In the status variable, censoring was entered as 0 and death as 1. Age, differentiation grade, residence, co-morbid disease, chemotherapy, histology type, weight, International Federation Gynecologist Obstetricians (FIGO) stage, tumour size, lymph vascular space invasion (LVSI), stage, metastatic number, and regional nodes were considered the independent variables that were assumed to influence the survival time of outpatients with breast cancer.

#### Operational definitions

The duration from the initial verified diagnosis of breast cancer and death is referred to as "survival" for breast cancer outpatients. Breast cancer "outpatients" are women whose diagnosis of breast cancer does not include pre-cancerous lesions. Early-stage patients with breast cancer are those in stages I and II, while late-stage patients with breast cancer are those in stages III and IV [26].

### Methods of data analysis

#### Descriptive statistics

When describing survival data, non-parametric techniques are utilised to compare the survival functions of two or more groups. To ensure uniformity in the usage of Laplace or lapse in this situation, Kaplan-Meier graphs would be utilized [27]. The information gathered from the registration book at the University of Gondar referral hospital was summarized using the frequency distribution table.

#### Survival data analysis

The crucial incident may not have been witnessed by research participants, therefore survival data is censored in that it does not give all the facts [28]. Survival analysis is well suited for breast cancer data sets which are frequently found in medical research due to the fact that follow-up studies in the medical profession can start at a certain observation time and can end before all experimental units have experienced an event.

#### Right censoring

Patient observation comes to an end just before the occurrence, to the right of the last recorded survival time. It was taken into account in this investigation because this type of censoring is widely accepted in survival analyses [29].

#### Comparison of survival function

The Kaplan-Meier graphs demonstrate that there may or may not be a difference in survival times between the groups of covariates considered. However, the log-rank test was used to assess whether or not the outpatient with breast cancer survival time in each covariate varied [30]. The hypotheses to be tested are:

- *H0:* The survival curves are the same for each.
- *H1:* The survival curves are different from one another.

#### Bayesian survival analysis

The Bayesian method for survival analysis is favoured above the frequentist approach in terms of the strength of the information the methodology may supply since it mixes probability data with previous knowledge about the distribution of the parameter. The Bayesian technique is more effective than the frequentist approach when analysing clinical data, making it a preferable choice for clinical researchers when selecting a data analysis strategy [31]; Some complex models simply cannot be estimated using conventional statistics; some people prefer the definition of probability; background data can be included in the analysis; and Bayesian statistics are not based on large samples (e.g., the central limit theorem), so large samples are not required for the maths to work. These are the major justifications for using Bayesian statistics. Additionally, by using the prior distribution, Bayesian statistics allow for the insertion of parameter uncertainty and the updating of this knowledge [32].

While treating the data as constant, the Bayesian approach regards the model's parameters as random variables, necessitating the definition of prior distributions for them. The Bayesian approach is notoriously difficult to use to fitting survival models, especially when complex censoring schemes are incorporated. The Gibbs sampler and other MCMC approaches can be used to reasonably easily fit complex survival models, and the availability of software greatly simplifies implementation [33]. The time-consuming nature of approximating the posterior and the convergence problem are two disadvantages of the MCMC techniques [22, 23]. 2009 also saw the introduction of the very flexible and speedy Integrated Nested Laplace Approximation (INLA) method [24].

*Prior Distribution* π(θ), The uncertainty of the parameter is stated using its probability distribution before the data are considered. It is a type of probability distribution known as a "prior distribution," which displays earlier information pertaining to the parameter of interest [33].

*Likelihood* L(θ/data), It is a likelihood function that determines how likely it is given the current parameters that the sample data will be observed. For a set of unknown parameters, it can be written as follows in the presence of right censoring:

$${\text{L}}\left(\uptheta /{\text{data}}\right)=\prod_{{\text{j}}=1}^{{\text{n}}} [{\text{f}}{\left({{\text{t}}}_{{\text{i}}}/{{\text{x}}}_{{\text{i}}};\uptheta \right)}^{{\updelta }_{{\text{i}}}}*{\text{S}}{\left({{\text{t}}}_{{\text{i}}}/{{\text{x}}}_{{\text{i}}};\uptheta \right)}^{{1-\updelta }_{{\text{i}}}}]$$

Where ${\updelta }_{{\text{i}}}$ is the censoring indicator (1=death and 0=censored) and S $\left({{\text{t}}}_{{\text{i}}}/{{\text{x}}}_{{\text{i}}};\uptheta \right)$ and $f\left({{\text{t}}}_{{\text{i}}}/{{\text{x}}}_{{\text{i}}};\uptheta \right)$ are the survival distributions and probability density respectively [34].

A likelihood comprises information about model parameters based on the observed data, while a prior gives information about model parameters from before the observed data was observed. The posterior distribution combines the prior distribution and likelihood using the Bayes rule. The likelihood function as a whole is multiplied by the prior distribution across all parameters, L(θ/data), to produce it [33]. Given by

$${\text{Posterior}}=\frac{Likelihood * prior }{\int \mathrm{Likelihood }*\mathrm{ prior d\theta }}$$

Assuming that θ is a random variable and has a prior distribution denoted by π(θ), then posterior distribution, π(θ/X), of θ is given by:

$$\uppi (\uptheta /{\text{X}})= \frac{{\text{L}}({\text{X}}/\uptheta )*\uppi (\uptheta ) }{\int {\text{L}}({\text{X}}/\uptheta )*\uppi (\uptheta )\mathrm{ d\theta }}$$

Evidently, π(θ/X) contains contributions from both the observed data through L(X/θ) and the prior knowledge quantified by π(θ) because it is proportional to the likelihood multiplied by the prior, π(θ/X) ∼ L(X/θ). Parametric survival models (exponential, weibull, log-normal, and log-logistic) are essential to Bayesian survival analysis because many Bayesian research are actually conducted using them. Simple to use modelling and analysis techniques are provided by parametric modelling [33].

#### Integrated nested Laplace approximation method

Through the use of the Integrated Nested Laplace Approximation (INLA) technique, the parameters of the Bayesian parametric survival models were established. Survival research frequently makes use of latent Gaussian models. In accordance with [24], INLA determines the posterior margins for each model component; the posterior expectations and standard deviations can then be determined from these. The integrated nested Laplace approximations may be applied to the latent Gaussian model of the survival models. Furthermore, INLA generates survival model-compatible posterior marginal approximations that are both extraordinarily speedy and incredibly exact [35], using novel Laplace approximations and powerful numerical approaches. R-INLA can be used in the same way as other R functions and acts as the INLA interface. You can obtain the INLA software and the R package for INLA for free at (http://www.r-inla.org).

#### Bayesian model selection criterion

We could choose to use the Deviance Information Criteria to compare Bayesian parametric survival models (DIC). It is best to use the model with the lowest DIC value [36]. The Watanabe Akaike Information Criteria (WAIC) [37], a different choice, offers a criterion utilising a more extensively Bayesian framework [21]. Claims that the DIC is outclassed by the WAIC.

#### Bayesian model diagnostics

The two most common techniques for assessing the goodness of fit are the Bayesian Cox-Snell residual plot and the Predictive Distribution. Model verification and appropriateness are key considerations in models for survival data. The residuals' Bayesian representation is seen in the Bayesian analysis employed [38].

## Results

### Descriptive statistics

The University of Gondar Referral Hospital in Gondar, Ethiopia, treated 382 patients with breast cancer at least twice between May 15, 2016, and May 15, 2020. These patients provided the study's data. The results of the categorical predictor variables for breast cancer patients are shown in Table 1 below. Accordingly, out of 382 outpatients, 148 (38.7%) died, according to statistics acquired from the University of Gondar referral hospital, and the remaining 234 (61.3%) were censored as outpatients. 219 (57.3%) of the 382 breast cancer outpatients had a literate level of education. A sample of 382 breast cancer outpatients included 155 (40.6%) who lived in rural areas. Of the 322 breast cancer outpatients, 102 (26.7%) had tumours that were 4 cm or larger. 161 (42.1%) of the 382 breast cancer outpatients had an early stage of the disease. 183 (47.9%) of 382 breast cancer outpatients had no co-morbid disease. Invasive lobular histology was present in 142 (37.2%) of the 382 breast cancer outpatients. A total of 60 (15.7%) of the 382 breast cancer outpatients had an invasive medullar histological type.

**Table 1:** Descriptive statistics of Categorical Variables of breast cancer, UoGRH, 2016-2020

Factor	Category	survival status		Total (%)
		Censored 234(61.3%)	Event 148(38.7%)	
Residence	rural	88 (23%)	67(17.5%)	155(40.6%)
	Urban	146(40.7%)	81(20.5%)	227(59.4%)
Educational level	literate	141(36.9%)	78(20.4%)	219(57.3%)
	Illiterate	93(24.3%)	70(18.3%)	163(42.7%)
Co-morbid disease	No	142(37.2%)	41(10.7%)	183(47.9%)
	Yes	92(24.1%)	107(33.2%)	199(52.1%)
Stage	early	121(31.7%)	40(10.5%)	161(42.1%)
	Late	113(29.6%)	108(28.3%)	221(57.9%)
Histology type	Invasive lobular	70 (18.3%)	72 (18.8%)	142(37.2%)
	Invasive medullar	46 (12%)	14 (3.7%)	60(15.7%)
	Invasive ductal	118 (30.9%)	62 (16.2%)	180(47.1%)
LVSI	Yes	23 (6%)	55 (14.4%)	78 (20.4%)
	No	211 (55.2%)	93 (24.3%)	304 (79.6%)
Chemotherapy	No	29 (7.6%)	21 (5.5%)	50 (13.1%)
	Yes	205 (53.7%)	127 (33.2%)	332 (86.9%)
Grade	Grade 1	40 (10.5%)	32 (8.4%)	72 (18.8%)
	Grade 2	44 (11.5%)	42 (11%)	86 (22.5%)
	Grade 3	20 (5.2%)	12 (3.1%)	32 (8.4%)
	Grade 4	130 (34%)	62 (16.2%)	192 (50.3%)
Radiation	Beam Radiation	70 (18.3%)	72 (18.8%)	142(37.2%)
	Brachytherapy	46 (12%)	14 (3.7%)	60(15.7%)
	BRB	118 (30.9%)	62 (16.2%)	180(47.1%)
Regional nodes	negative	165 (43.2%)	36(9.4%)	201(52.6%)
	Positive	69 (18.1%)	112 (29.3%)	181(47.4%)
Metastasis number	0	169 (44.2%)	108 (28.3%)	277 (72.5%)
	1	17 (4.4%)	3 (0.8%)	20 (5.2%)
	2	8 (2.1%)	3 (0.8%)	12 (3.1%)
	>= 3	10 (2.6%)	4 (1%)	14 (3.7%)
FIGO stage	IA1	24 (6.3%)	7 (1.8%)	31 (8.1%)
	IA2	28 (7.3%)	8 (2.5%)	36 (9.4%)
	IB1	22 (5.8%)	7 (1.8%)	29 (7.6%)
	IB2	19 (5.0%)	7 (1.8%)	26 (6.8%)
	IIA	24 (6.3%)	12 (3.1%)	36(9.4%)
	IIB	19 (5.0%)	14 (3.7%)	33 (8.6%)
	IIIA	34 (8.9%)	29 (7.6%)	63(16.5%)
	IIIB	28 (7.3%)	30 (7.9%)	58 (15.2%)
	IV	36 (9.4%)	34 (8.9%)	70 (18.3%)
Tumor size (cm)	<4 cm	185 (48.4%)	95 (24.9%)	280 (73.3%)
	>=4 cm	49 (12.8%)	53 (13.9%)	102 (26.7%)

*FIGO* International federation of gynecologist obstetricians, *UoGRH* University of Gondar referral hospital

Table 2 displays the patient outcomes for age and weight as continuous baseline values. The average baseline weight of the outpatients was 49.8 kg, and their standard deviation was 6.89 kg. The baseline average age was 44.4 years, with a standard deviation of 6.09 years. The minimum entrance age for the outpatient clinic for breast cancer is 18.

**Table 2:** baseline traits of a continuous variable of breast cancer patients*,* UoGRH, 2016-2020

Variables	N	Minimum	Maximum	Mean	Stand.deviation
Weight in K.g	382	36	60	49.8	6.89
Age in year	382	18	78	44.4	6.09

### Checking cox PH assumption and variable selection

The *p*-values for stage, co-morbid illness, and LVSI are lower than the typical level of significance (5%). As a result, there is a statistically significant association between Schoenfeld residuals and survival time, and a global test was significant (*p*-value = 0.046), as shown in Table 3, demonstrating the invalidity of the Cox-PH model assumption for the breast cancer data set.

**Table 3:** Cox model's proportional hazard assumption for breast cancer patients, UoGRH, 2016-2020

Covariate	chi-square	Df	*p*-value
Education	0.67	1	0.587
Residence	0.32	1	0.379
Base line weight	0.76	1	0.820
Tumor size	1.76	1	0.412
Grade	0.72	3	0.078
Histology type	0.80	1	0.149
Chemotherapy	0.87	1	0.331
Radiation	0.59	2	0.231
Regional nodes	0.53	1	0.09
Metastasis number	0.79	3	0.13
Stage	0.88	1	0.037
FIGO stage	3.82	8	0.267
LVSI	0.32	1	0.049
Co-morbid disease	0.16	1	0.038
GLOBAL	6.95	31	0.046

### Multivariable analysis of Bayesian AFT model using INLA methods

As shown in Table 3, the Cox-PH model's underlying assumption was false for the breast cancer data set; hence, parametric AFT models were used in its place. We assume that all of these coefficients have a normal prior with a mean of 0 and a variance of 1000 given that that β= (β_0_, β_1_,... β_p_)′ denotes the vector of covariate coefficients considered for analysis, 0 represents the intercept, and p represents the number of covariates (*p* = 15). We suppose that the Weibull, log-normal, and logistic distributions were subjected to an application of a gamma prior with shape parameter 1 and inverse scale parameter 0.001 to them.

The models that employ the breast cancer data set are contrasted in Table 4. To compare the efficacy of these multiple models using DIC and WAIC, the model with the lowest value and the best fit was chosen. Because the bold values are the least, it was concluded that the Bayesian Weibull AFT model (DIC = 1499.93; WAIC = 1498.67) was the best option for the survival time of outpatients with breast cancer among the available possibilities. The purposeful variable selection strategy was used to add the variables from the univariate to the multivariable Bayesian Weibull AFT model after choosing a suitable model. The model was then fitted using the estimated values of the important covariates.

**Table 4:** The comparisons of Bayesian AFT model using INLA methods, breast cancer patients, UoGRH, 2016-2020

**Distributions**	**DIC**	**WAIC**
Exponential	1603.92	1626.73
Log‑Normal	1590.20	1591.71
**Weibull**	**1499.93**	**1498.67**
Log-logistic	1529.91	1529.57

Bold values indicate better results than other filtering method

The Bayesian Weibull AFT model's final results, presented in Table 5, show that a variety of variables, including stage, co-morbid disease, grade, weight, FIGO stage, histology type, radiations, LVSI, chemotherapy, regional nodes, metastasis number, and tumour size, have statistically significant effects on the survival times of outpatients with breast cancer.

**Table 5:** Bayesian AFT model using INLA methods Results of cervical cancer outpatients, UoGRH, 2016-2020

Parameter	Pmean	Sd	median	Credible Interval	Mode	Kld
Intercept	6.34	0.16	5.71	[5.46, 6.52]^a^	4.4	0
Baseline weight	-0.21	0.15	-0.41	[−0.62, −0.15]^a^	-0.39	0
Stage (ref=early)						
Late	-0.41	0.14	-0.21	[−0.42, −0.03]^a^	-0.20	0
education(ref=literate)						
illiterate	-0.28	0.13	-0.18	[−0.32, 1.13]	-0.17	0
Histology type(ref= Invasive ductal)						
Invasive lobular	-0.299	0.05	-0.28	[−0.47, −0.14]^a^	-0.27	0
Invasive medullar	-0.19	0.02	-0.22	[−0.29, −0.08]^a^	-0.21	0
Metastasis number (ref= 0)						
1	-0.42	0.18	-0.27	[-0.72, -0.08]^a^	-0.22	0
2	-0.47	0.16	-0.39	[-0.71, -0.10]^a^	-0.32	0
>=3	-0.55	0.13	-0.41	[-0.87, -0.10]^a^	-0.32	0
Comorbid disease (ref=No)						
Yes	-0.42	0.11	0.31	[−0.51, −0.16]^a^	-0.30	0
Differentiation grade (ref=1)						
Grade 2	-0.67	0.10	-0.27	[-0.72, -0.08]^a^	-0.29	0
Grade 3	-0.43	0.16	-0.39	[-0.71, -0.09]^a^	-0.30	0
Grade 4	-0.33	0.10	-0.34	[-0.84, -0.10]^a^	-0.36	0
Residence (ref= urban)						
Rural	-0.38	0.33	-0.28	[−0.42, 2.63]	-0.27	0
Regional nodes (ref= negative)						
positive	-0.53	0.05	0.32	[-0.81, -0.26]^a^	-0.30	0
Radiation (ref=Beam Radiation)						
Brachytherapy	-0.28	0.13	-0.18	[-0.32, 1.13]	-0.17	0
BRB	-0.48	0.10	-0.31	[-0.63, -0.06]^a^	-0.32	0
Chemotherapy (ref= No)						
Yes	-0.32	0.06	-0.41	[-0.53, -0.25]^a^	-0.49	0
FIGO (ref=IA1)						
IA2	-0.44	0.10	-0.37	[-0.72, -0.09]^a^	-0.39	0
IB1	-0.48	0.18	-0.40	[-0.71, -0.08]^a^	-0.45	0
IB2	-0.52	0.15	-0.43	[-0.70, -0.02]^a^	-0.41	0
IIA	-0.57	0.13	-0.47	[-0.78, -0.04]^a^	-0.48	0
IIB	-0.61	0.10	-0.48	[-0.78, -0.14]^a^	-0.43	0
IIIA	-0.66	0.17	-0.43	[-0.71, -0.14]^a^	-0.46	0
IIIB	-0.70	0.15	-0.44	[-0.83, -0.14]^a^	-0.46	0
IV	-0.76	0.13	-0.51	[-0.87, -0.10]^a^	-0.42	0
Tumor size (ref= <4 cm)						
>=4 cm	-0.43	0.15	-0.27	[-0.46, -0.05^a^	-0.25	0
LVSI (ref= No)						
Yes	-0.45	0.10	-0.35	[-0.57, -0.15]^a^	-0.31	0
Tau parameter(log-normal)	3.5	0.47	4.8	[3.29, 5.27]^a^	4.15 _	

^a^indicated statistically significant at 5%. *Pmean* Posterior Mean, *Sd* Standard deviation, *Kld* Kullback-Leibler divergence

The acceleration factor from Table 5 and a 95% believable range of Bayesian accelerated failure time estimated values were used to understand the resulting model. The equation γ= [exp($\widehat{\beta }$) ]= [exp(posterior mean)] can be used to obtain the estimated acceleration factor.

The acceleration factor for breast cancer outpatients who lose one kilogram is predicted to be 0.81, with a 95% CrI of -0.62 to -0.15, by observing weight and adjusting for other factors. This means that their anticipated survival time will be 19% shorter than that of outpatients who gain one kilogram.

The estimated acceleration factor for breast cancer outpatients with invasive lobular histology is 0.75, with a 95% CrI of -0.47 to -0.14, keeping the impact of other covariates constant. Therefore, the length of patient survival time was significantly impacted by breast cancer outpatients who have invasive lobular histology type. Therefore, invasive lobular histology was associated with a 25% lower predicted survival time for breast cancer outpatients than invasive ductal histology.

The estimated acceleration factor for breast cancer outpatients with a late stage is expected to be 0.66 with a 95% CrI of -0.42 to -0.03, holding the impact of other factors constant. Therefore, the late stage had a large impact on the patient's period of survival. Therefore, compared to outpatients with early-stage breast cancer, the anticipated survival duration for those with late-stage breast cancer was 34% shorter.

The calculated acceleration factor for breast cancer outpatients with co-morbid disease, assuming all other variables remain constant, is 0.66, with a 95% CrI range of -0.51 to -0.16. Therefore, the survival of time breast cancer outpatients was significantly impacted by co-morbid disease. As a result, the anticipated survival time for breast cancer outpatients with co-morbid disease was 34% lower than it was for those with no comorbid disease.

With a 95% CrI of -0.72 to -0.08, -0.71 to -0.10, and -0.87 to -0.10, respectively, the estimated acceleration factors for breast cancer outpatients with metastases numbers 1, 2, and >=3 are projected to be 0.66, 0.63, and 0.58. As a result, the number of metastatic sites had a big impact on how long women survived as breast cancer outpatients. Therefore, compared to breast cancer outpatients with metastases 0, the anticipated survival durations for those with metastases 1, 2, and >=3 were 34%, 37%, and 42% shorter, respectively.

With a 95% CrI of -0.72 to -0.08, -0.71 to -0.09, and -0.84 to -0.10, respectively, the estimated acceleration factor for breast cancer outpatients with grades 2, 3, and 4 is estimated to be 0.57, 0.65, and 0.72 while controlling for other covariates. As a result, the grade had a big impact on how long women survived as outpatients with breast cancer. Therefore, compared to breast cancer outpatients in grade 1, the anticipated survival durations for those in grades 2, 3, and 4 were 43%, 35%, and 28% shorter, respectively.

While taking into consideration other factors, the calculated acceleration factor for outpatients with breast cancer who have illiterate educational levels is 0.76 with a [95% CrI of -0.32, 1.13]. The 95% CrI for the acceleration factor in illiterate outpatients with breast cancer did include one, indicating that this factor does not significantly alter the outpatients' breast cancer survival time.

The estimated acceleration factor for breast cancer outpatients receiving chemotherapy is 0.73, with a 95% CrI of -0.53 to -0.25, keeping the impact of other parameters constant. Chemotherapy therefore had a considerable impact on the duration of survival for breast cancer outpatients. Therefore, the anticipated survival time for breast cancer outpatients receiving chemotherapy was 27% lower than for breast cancer outpatients not receiving chemotherapy.

With a 95% CrI of -0.63 to -0.06, the estimated acceleration factor for breast cancer outpatients receiving beam and brachytherapy radiation is expected to be 0.62, keeping the impact of other parameters constant. Therefore, the survival time of breast cancer outpatients was significantly impacted by the beam and brachytherapy radiation combination. Therefore, compared to breast cancer outpatients without beam radiation, the anticipated survival duration for those who received a combination of beam and brachytherapy radiation was 38% shorter.

Keeping other factors constant, the estimated acceleration factor for breast cancer outpatients with FIGO stages IA2, IB1, IB2, IIA, IIB, IIIA, IIIB, and IV is 0.64, 0.62, 0.59, 0.57, 0.54, 0.52, 0.50, and 0.47, with a 95% CrI of -0.72 to -0.09, -0.71 to -0.08, -0.70 to -0.02, -0.78 to -0.04, -0.78 to -0.14, -0.71 to -0.14, -0.83 to -0.14, and -0.87 to -0.10, respectively. As a result, the FIGO stage had a big impact on how long patients survived as outpatients with breast cancer. Therefore, compared to breast cancer outpatients with FIGO stage IA1, the anticipated survival durations for those with FIGO stages IA2, IB1, IB2, IIA, IIB, IIIA, IIIB, and IV were 36%, 38%, 41%, 43%, 46%, 48%, 50%, and 53% shorter, respectively.

In the Bayesian Weibull AFT model, Table 5 demonstrates that all significant parameters had Kullback-Leibler divergence values of 0, which are tiny and indicate that the posterior distribution was closely approximated by a normal distribution. A Laplace approximation that has been simplified was the quickest and most efficient method.

#### Bayesian model diagnostic

**Fig. 1:**
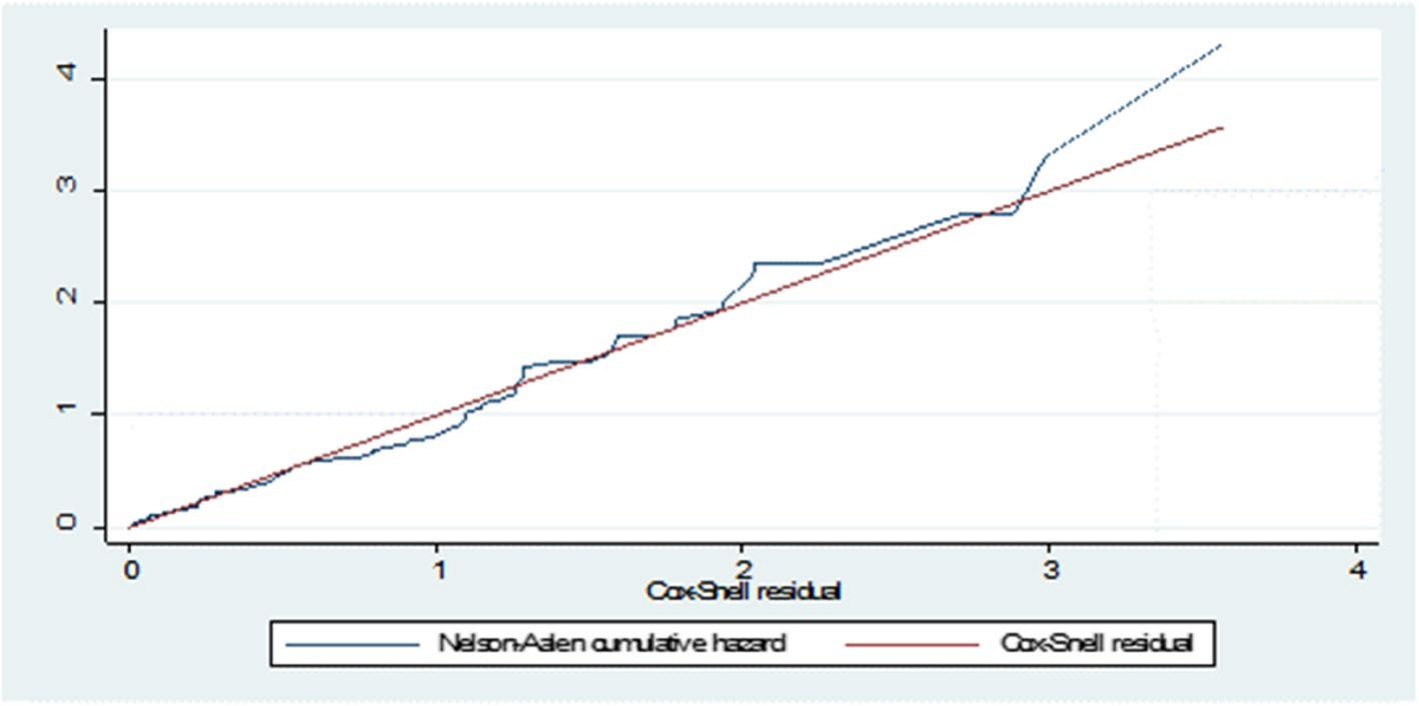
Bayesian Cox-Snell residual plots for baseline distribution and Cox-Ph that were used to fit the breast cancer patients, UoGRH, 2016-2020

The Bayesian Cox-Snell residual plot for the Bayesian Weibull AFT model was closest to the line through the origin, and the Cox-Snell residual plot versus the cumulative hazard function of residuals was essentially a straight line with slope 1. The Bayesian Weibull AFT model, out of the five models, best suited the breast cancer data set, as shown by the Bayesian Cox-Snell residual plots in Fig. 1. Further evidence was provided by the graphic indicating the Bayesian Weibull model adequately describes the breast cancer data set. Given that the total number of observations linked to failure flags in the breast cancer data set equals zero and that the conditional predictive ordinate values are noticeably smaller (by an order of magnitude) than the others, the observed values are surprising in terms of the Bayesian Weibull model. Incorporating a 95% confidence interval into the plots is done by looking at the posterior densities for the parameters in the breast cancer data set that had a normal distribution. As shown in Table 5, all relevant parameters in the Bayesian Weibull AFT model have a kullback-Leibler divergence (kld) value of zero, which is a diagnostic used to assess the precision of the INLA approximation.

## Discussion

This study's main objective was to assess the variables that contributed to the breast cancer outpatients' longer survival times at the University of Gondar referral hospital. Exogenous hormone exposure from things like oral contraceptives, HRT, and dietary fat intake increases the risk of breast cancer [9, 10]. Based on information gathered from the University of Gondar referral hospital for this study, 148 (387%) patients died. This mortality rate exceeded that of research conducted in central Ethiopia, which showed a 9.8 per 100 patient-year mortality rate [39]. The co-morbid illnesses and advanced clinical stage of the study participant may be to blame for the greater fatality rate in our study. It is consistent with earlier studies [40], which show that having one or more co-morbidities considerably raises the death rate for breast cancer survivors. It's possible that the advanced state of the disease at diagnosis in our study contributed to the greater frequency of fatalities.

Applying INLA, Bayesian parametric survival models were used. However, the Cox-PH model's premise was broken. The Bayesian technique was utilised, and DIC and WAIC calculations were made, in order to assess the effectiveness of several AFT models [36, 37]. The Bayesian Weibull AFT model, one of the alternatives offered, provided the most accurate description of the breast cancer data set. Results from a previous study [41, 42] had similar effects.

However, the Bayesian Weibull AFT model results using the INLA method in this study demonstrate that stage, grade, co-morbid disease, weight, histology type, FIGO stage, radiation, chemotherapy, LVSI, metastatic number, regional nodes, and tumour size all significantly affect the survival time of outpatients with breast cancer. The results are consistent with research [41, 43, 44].

Therefore, the survival duration of outpatients with breast cancer was significantly correlated with the disease stage. Advanced cancer stages are strongly connected with lower breast cancer patient survival rates [45]. This study also demonstrated that breast cancer outpatients in the late stage had a 34% worse anticipated survival time than those with breast cancer in the early stage. Stage-IV breast cancer outpatients have a threefold higher risk of passing away than stage-I breast cancer outpatients, according to TASH research [46]. This outcome is in line with the findings of a systematic study that evaluated the survival of BC patients in high- and low-income nations. In LMICs, only 20–50% of breast patients appear in the early stages. The very long wait for a consultation, access restrictions, poor quality cancer care and treatment, unfavourable attitudes towards the disease and its treatments among patients and the community, faith in the efficacy of complementary and alternative therapies, and a lack of social networks for support could all contribute to a diagnosis at an advanced stage in LMICs [47, 48].

Most research conducted in the industrialised world demonstrates a correlation between advanced clinical stages of breast cancer and treatment wait times longer than three months [47]. In high-income nations, the majority of breast cancer patients are detected in stages I and II, in contrast to our analysis, where the majority of patients had locally advanced disease [47]. We discovered that people with advanced breast cancer have a 3.01-fold higher probability of dying than those with early-stage disease. The results are consistent with research from Mexico, Hawaii, the United States, Nigeria, and Uganda [49–52].

It is therefore evident that earlier presentation or down staging of breast cancer will have a significant impact on survival rates. To ensure that patients benefit from early detection and timely treatment, it is essential to identify the barriers and facilitators in the local context so interventions can be implemented that address them. Adjuvant treatment prolonged disease-free survival (DMFS) and overall survival (OS), and multivariate analysis of tumours with a diameter of 26 cm showed lower loco-regional recurrence-free survival (LRFS) and OS, according to studies on the prognosis and survival of patients with locally progressed breast cancer [53]. According to our findings, the anticipated survival time for breast cancer outpatients with tumours measuring more than or equal to 4 cm was 35% shorter than it was for those with tumours measuring less than 4 cm. This outcome is consistent with [45].

According to the study's findings, concomitant illness was a strong predictor of how long breast cancer outpatients would live. In comparison to breast cancer outpatients without co-morbid disease, the estimated survival time for those with co-morbid disease was 34% shorter. This outcome is consistent with [47].

LVSI is reportedly a significant factor in the patients' poor prognosis for early-stage breast cancer. It is indicative of a patient's chance of survival to compare diffuse lymphatic involvement (diffuse 1.VSI) to focal or non-focal lesions [2, 27, 54]. In our study, the survival times for outpatients with LVSI were short.

In our study, the survival time decreased as the FIGO stage for outpatients rose. The FIGO stage is widely used therapeutically to predict the prognosis of patients with breast cancer, and the use of a nomogram could lessen the variation brought on by different treatments and socio-demographic statuses [55]. Patients with stage IV breast cancer have a 1.82-times higher probability of dying than those with stage I breast cancer. Similar to this, it has been commonly reported that breast cancer patients with stage III disease have a drastically reduced chance of surviving the disease [2].

For the Cox-PH, log-normal, Weibull, exponential, and log-logistic models, cumulative hazard graphs for the Bayesian Cox-Snell residuals were produced, as shown in Fig. 1. The plots for the Bayesian Weibull model were nearer the line, suggesting that it was the model that best suited the breast cancer data set and was in line with the earlier study by [42]. The model in this work was also evaluated using probability integral transformations and conditional predictive indices. Prior to doing an adequacy check using graphical methodologies, it can be necessary to verify whether the typical numerical mistake occurred during the computation of the conditional predictive ordinate. The numerical concerns of the breast cancer data set were unconnected since no failures were discovered and the overall number of conditional predictive failures was zero. The histogram and scatter plot of the probability integral transform revealed a fair predictive distribution that closely reflects the observed data, and the plots of predicted residual-based values were mostly evenly distributed with a few outliers that deviated from the norm. This conclusion was further supported by [35, 41, 44] and [56]'s follow-up studies.

The posterior density map for the parameters was shown to be normally distributed in the Bayesian Weibull AFT model diagnostic charts, which also included a 95% confidence interval. Similar to this, the diagnostic Kullback-Leibler divergence is used to determine how accurate the INLA approximation is. In this investigation, the Bayesian Weibull AFT model's kld values were set to zero for all pertinent parameters. This demonstrates the superior accuracy and speed of the Bayesian Weibull AFT model [56] and [35] lend support to these conclusions.

### Conclusions and recommendation

The survival periods of outpatients with breast cancer who received therapy for at least two visits at the University of Gondar referral hospital were taken from a data set for this study. Several parametric models with baseline distributions, including the log-logistic, Weibull, exponential, and log-normal, were outperformed by the Bayesian Weibull AFT model. DIC and WAIC show that these models have strong predictive performance. They could therefore be viewed as trustworthy tools for determining prognosis, which is essential for raising the patient's likelihood of survival. The results of this study indicated that the survival times of breast cancer outpatients were shortened by weight loss, the presence of comorbid disease, cell histology type, a large tumour size, an increase in the International Federation of Gynaecologists and Obstetricians stage, an increase in the number of metastases, an increase in grade, positive regional nodes, lymphatic vascular space invasion, and late stages of cancer. We advise healthcare professionals to begin treatment early for all breast cancer outpatients in order to raise the survival rates of all breast cancer outpatients and strengthen routine breast cancer screening programmes for high-risk women, such as those with large tumour sizes and breast cancer patients with co-morbid disease.

#### Strength of the study

The strength of our study is that the practical applications of cognitive behavioural therapy have had high success rates. The current approach (including controls and analysis protocols) is appropriate for the objective of the study.

#### Limitations of the study

Using secondary data may have introduced biases or inaccuracies, which is one of the study's acknowledged limitations. An further limitation was the lack of some variables in the medical records; this can be considered in future research to enhance the study.

## Data Availability

On reasonable request, the corresponding author will provide the datasets used and/or analysed during the current work.

## Abbreviations

AFT: Accelerated Failure Time
CIN: Cervical intraepithelial neoplasia
Cox PH: Cox Proportional Hazard
DIC: Deviance Information Criteria
INLA: Integrated Nested Laplace Approximation
KLD: Kullback–Leibler Divergence
MCMC: Markov chain Monte Carlo
OPD: Out-Patient Department
TASH: Tukir anbsa specialized hospital
UoGRH: University of Gondar referral hospital
WAIC: Watanabe Akaike Information Criterion
WHO: World health organization
