# Correction to: Assessing the survival time of women with breast cancer in Northwestern Ethiopia: using the Bayesian approach

**DOI:** 10.1186/s12905-024-02987-3

**Published:** 2024-03-06

**Authors:** Chalachew Gashu, Aragaw Eshetie Aguade

**Affiliations:** 1 Department of Statistics, College of Natural and Computational Science, Oda Bultum University, Chiro, Ethiopia; 2 Department of Statistics, College of Natural and Computational Science, University of Gondar, Gondar, Ethiopia

**Correction to: BMC Women’s Health (2024) 24:1**

10.1186/s12905-024-02954-y.

Following publication of the original article [1], author’s name Aragaw Eshetie Aguade was incorrectly written as Aragaw Ehetie.

The original article has been corrected.
